# Control systems for membrane fusion in the ancestral eukaryote; evolution of tethering complexes and SM proteins

**DOI:** 10.1186/1471-2148-7-29

**Published:** 2007-02-23

**Authors:** V Lila Koumandou, Joel B Dacks, Richard MR Coulson, Mark C Field

**Affiliations:** 1 Department of Pathology, University of Cambridge, Tennis Court Road, Cambridge, CB2 1QP, UK; 2 The European Bioinformatics Institute, Wellcome Trust Genome Campus, Hinxton, Cambridge, CB10 1SD, UK

## Abstract

**Background:**

In membrane trafficking, the mechanisms ensuring vesicle fusion specificity remain to be fully elucidated. Early models proposed that specificity was encoded entirely by SNARE proteins; more recent models include contributions from Rab proteins, Syntaxin-binding (SM) proteins and tethering factors. Most information on membrane trafficking derives from an evolutionarily narrow sampling of model organisms. However, considering factors from a wider diversity of eukaryotes can provide both functional information on core systems and insight into the evolutionary history of the trafficking machinery. For example, the major Qa/syntaxin SNARE families are present in most eukaryotic genomes and likely each evolved *via *gene duplication from a single ancestral syntaxin before the existing eukaryotic groups diversified. This pattern is also likely for Rabs and various other components of the membrane trafficking machinery.

**Results:**

We performed comparative genomic and phylogenetic analyses, when relevant, on the SM proteins and components of the tethering complexes, both thought to contribute to vesicle fusion specificity. Despite evidence suggestive of secondary losses amongst many lineages, the tethering complexes are well represented across the eukaryotes, suggesting an origin predating the radiation of eukaryotic lineages. Further, whilst we detect distant sequence relations between GARP, COG, exocyst and DSL1 components, these similarities most likely reflect convergent evolution of similar secondary structural elements. No similarity is found between the TRAPP and HOPS complexes and the other tethering factors. Overall, our data favour independent origins for the various tethering complexes. The taxa examined possess at least one homologue of each of the four SM protein families; since the four monophyletic families each encompass a wide diversity of eukaryotes, the SM protein families very likely evolved before the last common eukaryotic ancestor (LCEA).

**Conclusion:**

These data further support a highly complex LCEA and indicate that the basic architecture of the trafficking system is remarkably conserved and ancient, with the SM proteins and tethering factors having originated very early in eukaryotic evolution. However, the independent origin of the tethering complexes suggests a novel pattern for increasing complexity in the membrane trafficking system, in addition to the pattern of paralogous machinery elaboration seen thus far.

## Background

Intracellular transport and vesicle trafficking are fundamental processes that occur in nearly all eukaryotic cells. These processes are complex, requiring the activity of 5–10% of the proteome in *Saccharomyces cerevisiae *and likely a similar commitment in most other systems. It has been suggested that the origin of the membrane trafficking machinery was a key innovation for creation of the eukaryotic cellular state [1-3], and the presence of this system clearly distinguishes modern eukaryotes from prokaryotic organisms. Regardless of the precise mode by which membrane transport occurs, for example vesicle mediated or compartmental maturation models, a crucial aspect of the membrane trafficking process is how specificity of membrane fusion is determined.

It was initially proposed that vesicle fusion compatibility was encoded exclusively by coiled-coil SNARE proteins, which interact directly with the general Sec18/NEM-sensitive factor fusion system [4,5]. This model required that each transport vesicle contain a specific vesicle (V or R)-SNARE protein that interacts with cognate target (T)-SNAREs including the Qa-SNAREs (or syntaxins) and additional Q-SNARE proteins, which are present in the membrane of the target organelle. In all fusion reactions combinatorial pairing of the SNAREs was thought to provide specificity, but recent work has shown that SNARE pairing can be promiscuous and that additional factors, e.g. Rab family small GTPases, the regulatory syntaxin-binding (SM) proteins, and the tethering complexes, must also be involved [6-8]. With the exception of the tether complexes, these various factors all share the characteristic of being part of multigene families, and having distinct members of the family localised to, and participating in, transport steps at discrete subcellular compartments [9].

Analyses of SNARE [10-12] and Rab [13] sequences are consistent with each family having evolved from an ancestral gene, which then gave rise to the major SNARE and Rab gene families present in extant eukaryotes. As individual SNARE/Rab subfamilies are associated with distinct subcellular organelles, the most likely interpretation is that development of each new organelle was concurrent with the emergence of a novel SNARE or Rab paralogue. The gene duplications required to define the major compartments of the endomembrane system appear to have taken place before the last common eukaryotic ancestor (LCEA) arose; a similar model is also likely for the vesicle coat machinery [14].

**Figure 1:**
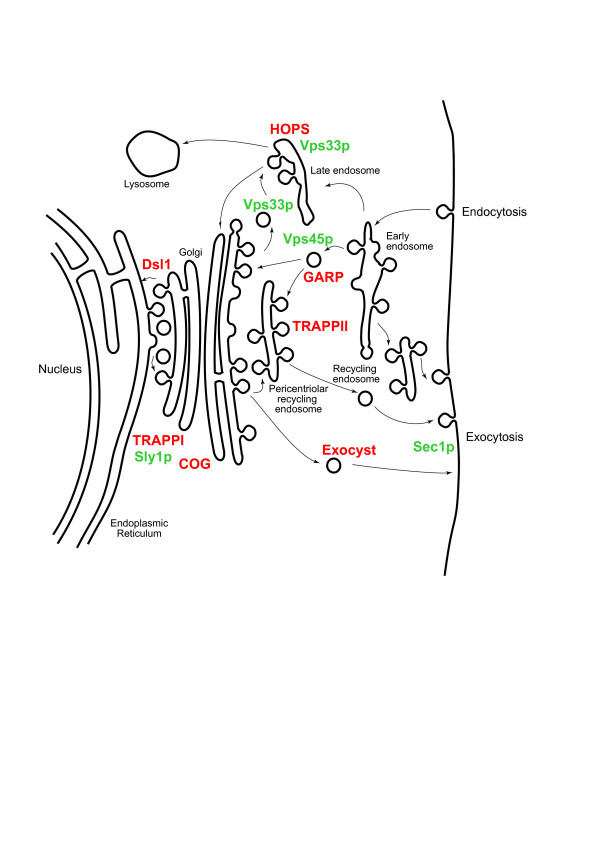
**Location and functions of multi-subunit tethering complexes and SM proteins in an idealised eukaryotic cell**. A generalised endomembrane system with only the major trafficking routes is shown, including the multiple recycling pathways that intersect with the Golgi complex and various endosomal subcompartments. The locations of tethering complexes discussed in this article are shown in red and of SM proteins in green. Note that the precise location is not always clear, for example the HOPS complex is known to function in late endocytic steps, but the functional and physical subdivision of the late endosomal population is not precise. Further, some factors may function in more than one locale. The figure is based on Figure 1 from Morgan *et al*., [64] with the permission of the authors.

The SM proteins bind syntaxins and regulate the *trans-*SNARE-SNARE interaction. As with SNAREs and Rabs, the SM proteins can be divided into protein subfamilies, each of which performs a similar function but at a specific and unique location within the cell (Figure 1). Surprisingly, although the SM proteins appear to all be derived *via *gene duplication, the different SM families bind syntaxins by distinct mechanisms [8]. Sly1p and Vps45p bind syntaxin 5 and syntaxin 16 respectively *via *the N-terminal domain of the SNARE in the open confirmation. Sec1p also interacts directly with its cognate syntaxin protein, but with the N-terminal domain of the SNARE folded over the C-terminal domain in a closed confirmation. By contrast, Vps33p binds its cognate SNARE indirectly as a part of the HOPS complex. These distinct modes of SNARE regulation by SM proteins have prompted speculation about how different mechanisms can result in essentially equivalent function and how such a situation could have arisen during evolution [15].

**Figure 2:**
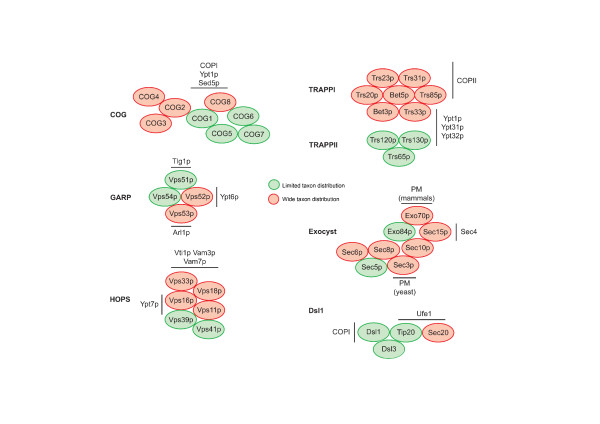
**Composition, evolutionary conservation and general structure of tethering complexes**. The individual subunits of each of the complexes are shown as ovals, arranged according to interaction data from many sources (including pull down, yeast two hybrid, direct structural visualisation and genetic data). For clarity, not all interactions are shown. For example, there is evidence to support interactions between COG 1 and COGs 3 and 4 and additional interactions within the exocyst complex. Individual subunits are colour-coded for evolutionary conservation; red designates a subunit that has wide taxon distribution, whilst subunits in green are absent from multiple taxa. Interactions with factors outside of the tethering complex are indicated in black – the line indicates the approximate interaction interface, if known. See Figure 4 and additional file 3 and the text for full details.

The tethering factors participate at the earliest stage in the approach of a vesicle towards a target membrane. The presence of such tethers has been suspected for some time [16], but whilst earlier work focused on potential roles of extended coiled-coil proteins as tethers, e.g. GM130 and Uso1, more compelling evidence for a predominant role for multiprotein complexes has recently emerged (reviewed in [7,17]). Knockout studies in *S. cerevisiae *[7,17,18] demonstrate stronger phenotypes for the components of many of these complexes than for the putative coiled-coil elements. Further evidence, again mainly from studies in *S. cerevisiae*, indicates interactions between these complexes and central components of the fusion apparatus, specifically SNAREs, Rab and ARF GTPases and coat proteins (Figures 1 and 2). In addition, the tethering factors may be isolated as stable complexes. Tethering complexes have differing combinations and numbers of subunits, with the individual complexes (COG, HOPS, TRAPPI, TRAPPII, DSL1, exocyst and GARP) acting at distinct subcellular locations (Figures 1 and 2).

Investigating membrane trafficking in organisms beyond the conventional experimental systems can identify conserved, presumably essential, membrane trafficking factors, as well as highlight the diversity of trafficking pathways amongst extant eukaryotes and uncover novel biology in specific lineages. From a combination of molecular and morphological data, six eukaryotic super-groups are now recognized [19,20], but molecular studies of intracellular transport systems have focused primarily on *S. cerevisiae *and metazoan taxa, both members of the Opisthokonta super-group. The relatively poor experimental tractability of many of the organisms within the additional eukaryotic super-groups poses a considerable challenge, but the availability of genome sequences from some of these taxa can facilitate rapid identification of factors in such systems.

Evolutionary investigation of the membrane trafficking machinery has revealed several features. Firstly, genomic, phylogenetic and cell biological evidence suggests that the LCEA possessed a complex endomembrane system. The major protein families known to be required for vesicle formation and fusion [9,21] were present very early on in eukaryotic evolution [22-24] and additional data support the presence of a full complement of membrane trafficking organelles [25,26]. We previously investigated the distribution of key components of major endocytic trafficking pathways, and found excellent conservation of both Rab and syntaxin genes plus components of the multivesicular body ESCRT system, suggesting that the major endocytic transport pathways are likely ancient [24,27]. However, evidence for substantial secondary losses of certain factors, for example Rab4, was also obtained, implicating secondary loss as a driver in the evolution of taxon-specific trafficking features together with emergence of novel functions in specific lineages [27]. Consistent with this pattern of loss or degeneration is a recent study of the N-glycosylation system, where multiple absences of genes responsible for the construction of the dolichol-PP-linked N-glycan precursor were detected [28], plus the independent loss of Golgi complex cisternal stacking in, at least, four major eukaryotic lineages [25].

An investigation in metazoa, yeast and streptophyte plants showed that the four SM protein families are separate and encompassed representatives of two eukaryotic super-groups, Opisthokonta and Viridiplantae [29]. However, further sampling of additional taxa is needed to properly address the distribution of the SM families and when these families originated. Similarly, a limited number of potential evolutionary relationships between the COG, GARP and exocyst tethering complexes have been described [17,30], based nearly exclusively on the presence of shared domains in yeast homologues of the complexes. Here we investigated the relationships and distributions of tethering factors and SM proteins across multiple representative taxa. In terms of deeper evolutionary relationships, we considered whether there was evidence for a common ancestral complex that gave rise to all of the extant tethering complexes, or independent evolution of each complex. The first model predicts a degree of sequence relatedness between the proteins comprising the different complexes. Further, we also considered the possibility that the tethering complexes may have arisen after the LCEA and therefore would display restricted taxon distribution. We find weak evidence for relationships between the complexes, explainable as the product of functional sequence constraints, and consistent with independent evolutionary origins. Furthermore, we find that the presence of SM proteins and the tethering complexes is wide spread, suggesting that they are ancient features of the membrane-trafficking machinery.

## Results and discussion

### Evolutionary relationships between and within tethering complexes

We chose to investigate, using BLAST approaches, the relationships between all identified subunits of the COG, exocyst, TRAPP, Dsl1, HOPS and GARP tethering complexes (Figure 2). These factors are involved in trafficking from the ER, through the Golgi complex and within the endosome/recycling system [31]. We initially addressed the evolutionary origin of the various tethering complexes to determine if there was evidence for a common origin or, alternatively, if evidence suggested an independent origin for each. Weak inter-complex relationships have been detected previously by PSI-BLAST algorithms demonstrating a distant relationship between some of the COG and exocyst subunits (COG3-Exo70p and COG8-Sec5p), and the Sec3p exocyst component and Vps52p GARP factor [17,30].

**Figure 3:**
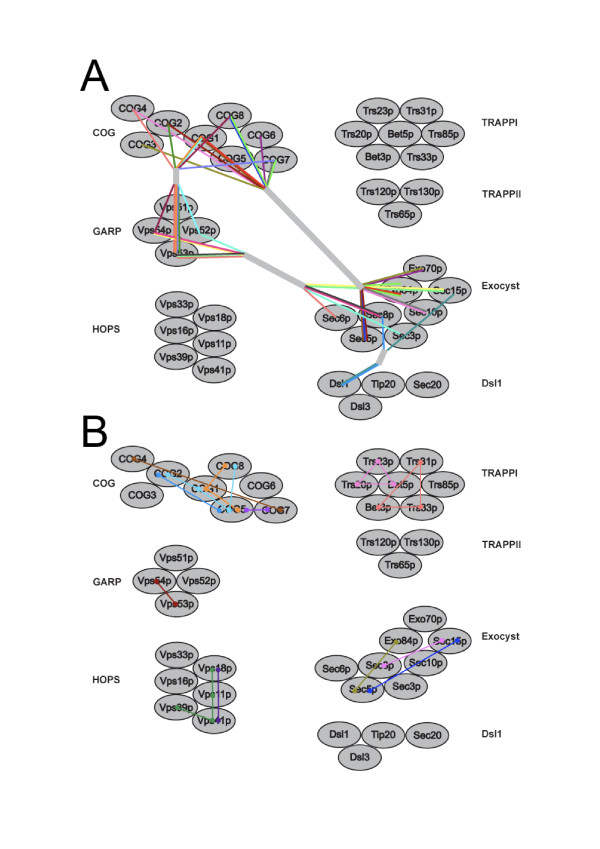
**Sequence relationships between tethering factor subunits**. Panel A, intercomplex relationships, panel B; intracomplex relationships. Sequence connections between subunits are colour-coded for clarity, and are based on PSI-BLAST hits, but the choice of colour is arbitrary. In panel A, the individual connections have been gathered together into grey ribbons between the complexes for clarity. Note that PSI-BLAST analyses retrieve multiple subunits of GARP, COG and exocyst in a reciprocal manner, while only two such associations are identified between Dsl1 and exocyst. HOPS and TRAPP are not interconnected with the other tethering complexes.

We analysed all of the *S. cerevisiae *tethering factors by PSI-BLAST against the non-redundant database and applied criteria for assessing homology as discussed in the methods, with a few numerical examples discussed below. The Vps52p-Sec3p relationship was confirmed, and we were able to detect further similarities between GARP components Vps53p and Vps54p and additional exocyst subunits, suggesting that a subfraction of the exocyst could be related by distant sequence similarity to GARP (Figure 3A). In addition, similarities between the GARP subunits Vsp52p, Vps53p and Vps54p and certain COG subunits were found, but many of these relationships are restricted to rather small regions of sequence spanning <200 amino acids. For COG, in addition to the relationship with GARP, we confirmed the previously reported COG3-Exo30p and COG8-Sec5p relationships, but we also found that COG1, 2, 4, 7 and 8 demonstrated some similarity to exocyst subunits, again of a limited nature. Significantly, the same subgroup of COG subunits demonstrates similarity with both GARP and the exocyst (COG1, 2, 4, 7, 8), but in contrast the exocyst subunits displaying relationships to GARP or COG were distinct. A relationship was also detected between ZW10, the metazoan equivalent of Dsl1, and two exocyst components. Interestingly, no intercomplex relationships were detected for the HOPS or TRAPP complexes. In no case was there evidence for an extensive relationship that encompassed the majority of the predicted protein sequence, or indication of a shared domain or overall architecture.

Intra-complex relationships were previously detected within an amphipathic helix present in several COG subunits (1, 2, 5 and 8), exocyst components (Sec5p, Sec8p and Exo84p) plus two of the GARP subunits (Vps53p and 54p), but the conservation is very weak, even between orthologues from taxonomically close organisms, e.g. *Homo sapiens *and *S. cerevisiae *COG2 (ldlC and Sec35p respectively) [30]. The significance of this latter relationship is unclear, especially given the compositional restriction imposed for a region presenting amphipathicity. We again used PSI-BLAST analysis to further investigate relationships within individual tethering complexes (Figure 3B). Multiple relationships between COG subunits were uncovered, although these excluded COG3 and COG6 that appear unrelated to the remaining COG components. Three putative relationships were found in exocyst, and which overall connects Sec3p, Sec5p, Sec15p and Exo84p. With the exception of Sec3p, these factors are also those that display connectivity to COG.

For the HOPS complex, four of the six subunits exhibit a degree of relatedness. As this complex does not appear to be related to other tethering complexes, possibly HOPS arose by repeated duplication of these related subunits, coupled with acquisition of the unique subunits, Vps16p and Vps33p. As Vps39p and Vps41p are less well retained across evolution (discussed below), this pattern of relatedness is also consistent with a degree of redundancy between these two subunits and Vps11p/Vps18p respectively. Vps33p contains a Sec1 domain, so this factor is essential for the correct interaction of HOPS with its cognate SNAREs, whilst Vps16p provides binding specificity for Ypt7p. Hence a likely minimal functional HOPS complex could be built from Vps11p, 16p, 18p and 33p, and such a configuration is present in several taxa.

Robust relationships were detected within the TRAPP complex for the presence of two families of proteins comprising the majority of TRAPP I; this has been reported previously [17] and suggests that TRAPP evolved from a simpler complex, likely consisting of single Bet3p and Bet5p isoforms. Bet3p, Trs33p and Trs31p all contain a Bet3 domain, whilst Trs20p, Trs23p and Bet5p have a sybindin domain in common. The Bet3 domain is a dimeric structure that forms hydrophobic channels and is likely responsible for membrane localisation [32], hence the presence of more than one Bet3p domain-containing protein is consistent with the stable interaction of TRAPP with the Golgi complex, and suggests a common origin for Bet3p, Trs31p and Trs33p. The presence of a sybindin domain in Bet5p, Trs20p and Trs23p also hints at more distant relationships with SNARE proteins as sybindin is a subset of the SNARE structural clan.

At face value, the above data could be taken as evidence for a common origin for the tethering complex subunits. However, with the exception of the TRAPPI complex, the relatively low E values for PSI-BLAST hits between the tether components (typically in the range 10^-4 ^to 10^-7^) only provide evidence of weak similarity bordering on the statistically insignificant. The alternative interpretation is that PSI-BLAST detected convergent evolution, i.e. the acquisition of common structural elements in otherwise disparate factors. This is commonly observed amongst coiled coil proteins, where the restriction on amino acid composition leads to highly similar sequences that have independent origins. In our PSI-BLAST analysis, hits of higher significance were found against coiled-coil regions of proteins that are clearly unrelated, for example myosin and Uso1p, than between tethering factors, suggesting the detection of limited sequence similarity that does not reflect an evolutionary relationship. By contrast, the clear homology between Trs23 and Bet5 in TRAPPI has a score of 10^-19^. Further, inspection of the similar regions identified by PSI-BLAST indicates that much of the similarity resides within sequence predicted to form coiled coil secondary structure; for example Vps53p-COG7, ZW10-Sec8p, COG1-COG8, Sec8-Vps54 and Vps54-Sec8p – in all cases the homologous region detected by PSI-BLAST is at the N-terminus, and the majority of this region is predicted as coiled coil (additional file 1). This finding is in agreement to that made by Whyte and Munro [17] for a more limited set of tether factors.

No evidence for any homology between TRAPP or HOPS and the other tether complexes was obtained, suggesting that the mechanisms by which these assemblies function may be rather distinct, beyond the simple provision of stabilisation of *trans-*SNARE complexes. The radically different levels of complexity of the tether complexes is also consistent with distinct modes of action, for example Dsl1 is limited to four subunits, whereas the full TRAPP complex comprises ten subunits. Hence despite a common mechanistic role, the precise molecular interactions underpinning distinct intracellular transport steps are potentially divergent. This may reflect interactions with distinct coat systems as well as other factors required to control and complete individual steps in vesicle transport.

Recent work has uncovered a further deep evolutionary relationship between the multiple coats responsible for protein sorting, membrane deformation and vesicle budding. For example, distant but clear homology exists between coatomer, clathrin and adaptin proteins, while a conserved architecture has recently been uncovered for proteins of the nuclear pore complex and the clathrin coat system [14,33]. Significantly, this latter relationship is not easily detectable by BLAST algorithms, and required secondary structure prediction and controlled proteolytic mapping to validate. However, the relationships differ further from those detected for the tether factors in being both more extensive, i.e. encompassing a greater proportion of the polypeptide, and more varied, i.e. including regions of the polypeptide with differing secondary structure. Hence they are of a more substantial nature than the tether complex similarities. We conclude that evidence for a common ancestry for the tether factors is not present, and it is most likely that these complexes have independent molecular origins, with convergent evolution of coiled-coil regions. Given the preponderance of coiled-coil proteins, including the SNAREs themselves, involved in vesicle fusion, the presence of these structural motifs within the tether complexes is perhaps not surprising.

### Tethering complex distribution across the eukaryotes

**Figure 4:**
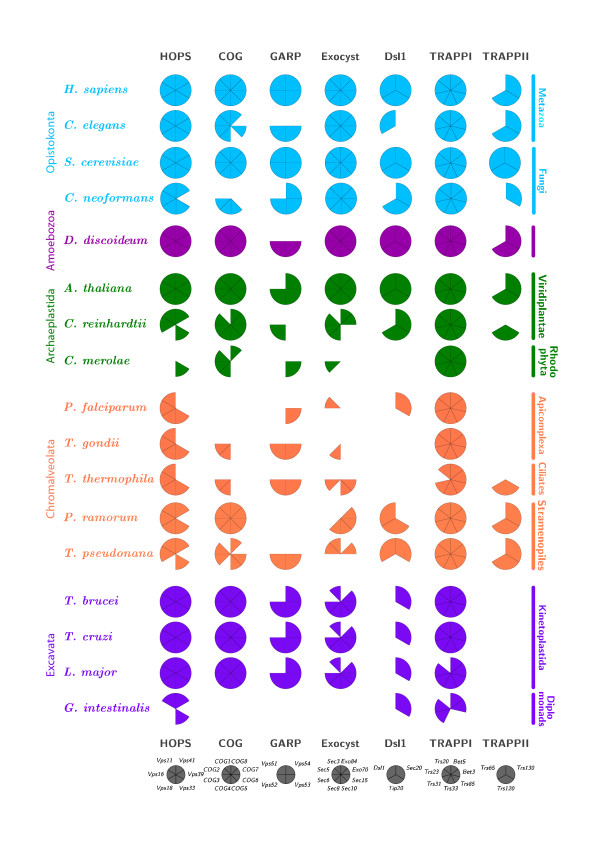
**Distribution of tethering complex subunits across representative eukaryotic taxa**. Data are based on BLAST results together with alignments – typically the *S. cerevisiae *or *H. sapiens *sequences were used as queries, as described fully in methods. Large taxon groupings are colour coded, and a key defining the factors represented by each sector is given at bottom. Filled sectors = an identification based on a clear reverse BLAST result and/or additional evidence through analysis of the sequence by Clustal [58]. Open sectors = not found. Individual BLAST results are provided in additional file 3. Note Dsl3/Sec39p has been omitted from this figure as it is only found in *S. cerevisiae*.

We probed a total of 17 genomes, with predicted protein sequences corresponding to 40 tethering factors, representing the entire tethering complex repertoire as known from *S. cerevisiae *and *H. sapiens *(Figure 4, additional file 3). The major feature that emerges from this analysis is the wide-spread taxonomic presence of the majority of the tethering complexes. Specifically, taking the most parsimonious view, the majority of taxa lacking a complex (or significant numbers of constituent factors) can be ascribed as either secondary loss or divergence of the sequence to such an extent as to be undetectable. Only the Dsl1 and TRAPPII complexes have a distribution that is equivocal. The evolutionary distributions of the individual complexes are considered below.

#### TRAPP

Evidence suggests that the seven subunit TRAPPI complex, involved in intra-Golgi anteriograde transport, has likely arisen via gene duplication, as Bet3p/Trs31p/Trs33p and Bet5p/Trs20p/Trs23p define subfamilies of the TRAPPI factors. Significantly, of these six subunits, only Trs33 is nonessential in yeast, as is the unrelated seventh subunit, Trs85. However, mutation in Trs20 leads to spondyloepyphesial dysplasia in humans [18], i.e. mutants are viable, indicating a lack of essentiality and therefore differential selection pressure for TRAPP subunits may exist in different taxa.

We observed near universal conservation of the TRAPPI complex across the taxa studied here (Figure 4, additional file 3), and therefore any gene duplication events clearly predate the speciation of the various eukaryotic lineages. The lack of three TRAPPI subunits from *Giardia intestinalis *may suggest that the TRAPPI complex is nonfunctional in *Giardia*, although interestingly two each of the Bet3 and Bet5 family are present. This divergent structure is potentially consistent with the novel aspects of trafficking in this organism where even basic organellar arrangements appear to be divergent from other eukaryotes [34].

The primary sequence structure of the individual TRAPPI subunits does appear to vary however, as several orthologues appear to have either extensions or deletions and differential levels of sequence conservation are seen; these likely represent emergence of species-specific functions. Bet3p is very highly conserved in terms of length throughout the eukaryotes, whilst Bet5p in *Arabidopsis thaliana *and *Plasmodium falciparum *contains a short N-terminal extension as compared to the *S. cerevisiae *and *H. sapiens *orthologues, and *Caenorhabditis elegans *has two isoforms, both of which have extensive C-terminal extensions (additional file 2).

For TRAPPII, Trs65 is unique to *S. cerevisiae*. The two remaining subunits, Trs120p and Trs130p, are more widely distributed, being found in several Opisthokonta taxa, as well as in the Amoebozoa, Viridiplantae, and stramenopiles, but TRAPPII is totally absent from the Apicomplexa, Excavata and *Cyanidioschyzon merolae*. Therefore the complete TRAPPII trimer is most likely a recent acquisition in *S. cerevisiae*, whilst Trs120p/Trs130p represent an ancestral form. Significantly Trs120 and Trs130 are essential in gene deletion experiments in *S. cerevisiae*, while Trs65 knockouts in yeast are viable and the effect on anteriograde transport is comparatively mild, consistent with the view that Trs120p/130p forms the minimal functional core [35].

#### Dsl1

This complex has a major role in Golgi to ER retrograde transport, and in *S. cerevisiae *all four subunits are essential. This essentiality contrasts with the low level of retention of Dsl1p and Dsl3p subunits across the eukaryotes; Dsl3p is unique to yeast whilst the core heterotrimer (Dsl1p/Tip20p/Sec20p) was only recovered from Opisthokonta, Amoebozoa and Viridiplantae groups (Figure 4, additional file 3). The Sec20 subunit demonstrates rather wider distribution than the holocomplex, also being found in the Excavata, stramenopiles and the Alveolata, suggesting that Sec20p can function independently of other Dsl1 subunits and consistent with its role as a Q-SNARE. A functional dimer of Dsl1p/Tip20p could retain both the COPI binding and Ufe1 T-SNARE interaction, linking coatomer and SNARE activity [36].

Organisms where none of the complex subunits are found, specifically *C. merolae*, *Toxoplasma gondii*, and *Tetrahymena thermophila*, suggest probable legitimate absence. Only Dsl1 is retained in *C. elegans*, a taxon closely related to the query *H. sapiens *sequence and with a reliable database implying true absence of the other subunits; fundamental differences in retrograde mechanisms amongst the metazoa, which have yet to be described in detail, may be indicated by this finding. These observations imply that the functions of all subunits can be dispensed with in certain lineages and may reflect the absence of a Dsl1 dependent Golgi to ER retrograde transport pathway in those systems.

#### Conserved oligomeric Golgi complex (COG)

The octameric COG complex mediates transport through the Golgi complex, and plays a particularly important role in maintenance of the N-glycosylation system, at least in mammalian cells. Of the COG subunits, only three, COG2, COG3 and COG4, are essential in knockout experiments in *S. cerevisiae*. Significantly, these three subunits are thought to form a subcomplex, based on yeast two-hybrid and co-immunoprecipitation studies [37].

COG is fully retained in *S. cerevisiae*, *H. sapiens*, the trypanosomatids, *A. thaliana*, *Dictyostelium discoideum *and *Phytophthora ramorum*. This distribution, together with the presence of partial COG complexes in additional taxa, is consistent with an ancient origin, together with some likely secondary losses of subunits (e.g. *T. thermophila *and *T. gondii*). Clearly, complete absence of COG can be tolerated, as neither the *P. falciparum*, nor *G. intestinalis *genomes encode detectable COG subunits, suggestive of legitimate loss of the complex in these taxa. Thus COG requirements are species-specific, as also shown by severe phenotype of COG1 mutants in mammalian cells [18], in contrast to no detectable phenotype in an RNAi knockdown in *Trypanosoma brucei *[38].

It is possible that the differential requirement for COG is due in part to the functionality required from the Golgi complex. Specifically, a role in maintaining the correct environment for N-glycan processing is clear from the phenotypes obtained in COG mutants in mammalian cells [18]. Amongst the taxa sampled here *P. falciparum *and *G. intestinalis *are unusual in that there is now excellent evidence for a highly diminished N-glycosylation machinery in these lineages, including a complete absence of mannosylation, and hence substrates for elaboration by galactosyl- or sialyltransferases [28]; the correlation between the absence of both mannose-containing N-glycans and the COG complex is highly suggestive of functional relatedness.

#### HOPS

This hexameric complex functions in endosome to vacuole/lysosome transport and is well conserved, despite the nonessentiality of the subunits in *S. cerevisiae*. Except for Vps39, this complex is almost fully conserved in most genomes. The exception is *C. merolae *where the complex is almost completely absent. Whilst Vps39 is poorly conserved at the sequence level, and it remains possible that for some taxa the open reading frame was not detected by BLAST, additional data also suggest that Vps39p and Vps41p are less important for HOPS function [39]. Vps11, 16, 18 and 33 are class B Vps mutants with severe phenotypes – Vps11p/16p/18p/33p appears to be the core complex with Vps39p and 41p (class C mutants with milder phenotypes) providing accessory subunits and function [39]. Indeed the Vps11p/16p/18p/33p complex can alternatively associate with Sec8p, instead of Vps39p/41p [39], providing a link to the membrane. Vps39p/41p interacts directly with Ypt7p (i.e. Rab7), and therefore the precise function of Rab7 may depend on the presence of Vps39p/41p. Because Vps39 is not found in the Chromalveolata genomes sampled here, this raises the possibility that this pathway is different in these taxa than in most other lineages.

#### GARP

GARP mediates an endosome to Golgi transport pathway. The complex is comparatively small, consisting of four subunits, none of which is essential in *S. cerevisiae*. Vps52p/53p/54p form a stable vesicle-associated complex [40], and Vps51p acts to tether this to the SNARE protein Tlg1p. The interaction may have little to do with function as ablation of the Vps51p-Tlg1p interaction has little effect *in vivo *in yeast [41] and presumably additional factors can recruit Vps52p/53p/54p to the membrane. This is also entirely consistent with the observation that Vps51 is the most sparsely distributed member of this complex, being restricted to taxa in the Opisthokonta lineage. Of the remaining subunits, Vps52p and Vps53p are the best retained across taxa. Interestingly the Vps52p/53p/54p complex is retained in higher plants and in trypanosomatids, indicating the presence of a retrograde transport system in these organisms that presumably actively returns material to the Golgi complex from the endosomal system. While the complex is not well conserved (Figure 4, additional file 3), the overall pattern of subunit occurrence argues that this is an ancient complex.

#### Exocyst

This complex directs exocytic vesicles to the plasma membrane and consists of two subcomplexes of three and five subunits each. All of the subunits are essential in *S. cerevisiae*. Significantly, different subunits appear to mediate interaction with the plasma membrane in mammals and yeast; in *S. cerevisiae *Sec3p binds Rho1p and the plasma membrane, while Sec15p binds the Sec4p GTPase on the vesicle membrane [42]. By contrast, in mammals Sec3p does not bind Rho1p or the plasma membrane; instead Exo70p may mark sites of exocytosis on the plasma membrane [43]. The full complex is present in the Opisthokonta, Amoebozoa and higher plants. However, the presence of at least some exocyst subunits in other taxa suggests that the exocyst is an ancient system. Our observations are consistent with a recent report describing the presence of Exo70p in land plants and other diverse eukaryotes [44]. In our analysis, no subunits are found in *G. intestinalis*, while only one is recovered in *C. merolae, P. falciparum *and *T. gondii*, suggesting that, in these systems, full exocyst function may be absent.

### Insights into taxon-specific functions

A number of interesting features are apparent from the data-set when considered by organism rather than by complex. Most dramatic is the absence of several complexes from a number of lineages. Specifically, no subunits were recovered in *G. intestinalis *for COG, GARP, exocyst or TRAPPII and only Sec20 of Dsl1 was found. Whilst there is the possibility of increased sequence divergence for this system, and hence failure to detect homologues that are in fact present, it is highly unlikely that this accounts for the extreme level of absence and is consistent with an earlier analysis sampling a wide range of endocytic functions [27] and with the somewhat unusual organisation of the endomembrane system in *Giardia *[34]. Also consistent with earlier work is the observation that *C. merolae *appears to possess only a complete TRAPPI and a possible minimal COG complex, with single subunits of a few of the other complexes. This feature may reflect both the small genome of this organism as well as the extreme environment (pH 2.0) that *C. merolae *exploits [45], suggesting a radically minimalised trafficking system [27]. The Apicomplexa may well lack several complexes, with good evidence for the presence of HOPS, and TRAPPI and only limited representation for the others. Given that we sampled two genomes from this group, and the patterns of subunit recovery are overall very similar, we consider this prediction to be robust. These observations indicate that mechanisms for transport through the Golgi complex, for exocytosis and for Golgi to ER trafficking are likely either mediated by novel factors, or alternatively are highly simplified in the Apicomplexa, in part reflected by a minimised N-glycosylation system in *P. falciparum *[28].

Perhaps less surprising is the high degree of conservation of the complexes throughout the Opisthokonta, Amoebozoa, Viridiplantae and the Kinetoplastida. However, significant variation in the complexes recovered even between comparatively closely related taxa does underscore the potential lability of the trafficking system. For example, in *Cryptococcus neoformans*, we were unable to recover by BLAST many subunits of both COG and TRAPPII, demonstrating a significant divergence between this system and the model yeast *S. cerevisiae*. The Kinetoplastida retain the majority of the complexes, lacking only Dsl1 and TRAPPII. Overall, the kinetoplastids are extremely similar to each other, emphasizing a strong retention of this machinery in these three parasites despite their very different life-cycles.

### SM proteins

Another major set of players in machinery encoding specificity of membrane fusion events are the SM proteins [8]. We, therefore, addressed the evolution of these components via comparative genomics and phylogenetics. Our BLAST search identified at least one putative homologue for each of the defined SM protein families in all of genomes examined (data not shown). While such evidence was used in a few cases, discussed below, to identify the various homologues, phylogenetic analysis was pursued in order to provide a more rigorous basis for annotation. An initial dataset was analyzed, composed of homologues of each of the SM protein families from representatives of the five sampled eukaryotic supergroups. From ML and ML-corrected distance analyses, the four SM protein families resolved with 100% support with both methods for the clades of Sly1p, Vps33p and Vps45p (data not shown). The clade of Sec1p was supported by bootstrap values of 81% and 99% with the two methods respectively. This provided preliminary evidence for the expansion of the four SM protein families prior to the divergence of most eukaryotic lineages.

**Figure 5:**
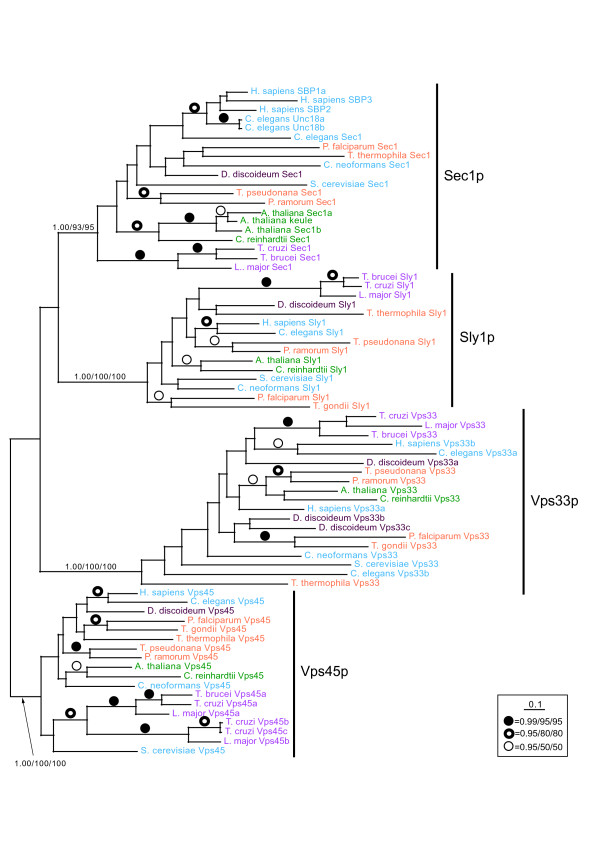
**Phylogenetic analysis of SM proteins from eukaryotes in the major sampled supergroups**. This analysis shows that the four SM protein families robustly form separate clades as shown by the bold support values and denoted by the vertical bars to the right of each clade. Support values are given in the order of Bayesian posterior probabilities/ML bootstraps/ML corrected distance bootstraps. That each family encompasses all of the sampled eukaryotic diversity is illustrated by the colour-coded taxon names, which follow the scheme of Figure 4. Support values for all nodes supported by 0.95 posterior probability and 50% bootstrap support or better, are illustrated symbolically. This analysis demonstrates that the SM protein families are ubiquitously found in the sampled eukaryotes and their evolution likely pre-dates the last common eukaryotic ancestor.

A second analysis included representative sequences from all taxa sampled. However, the resolution of this dataset was poor (data not shown) and the sequences from *G. intestinalis *and *C. merolae *represented divergent, and presumably rapidly-evolving, homologues. These were removed from the dataset and the resulting alignment was then analysed by Bayesian, ML and ML corrected distance methods. As in the preliminary analysis, there was very good support for the robust separation of clades representing homologues of Sec1p, Sly1p, Vps33p and Vps45p, and each clade contained representatives of each of the five sampled eukaryotic super-groups (Figure 5). Finally we aligned the most canonical sequences of each putative SM family from *G. intestinalis *and *C. merolae *(as predicted by BLAST) to an SM alignment with one representative of each supergroup for each paralogue family. In the case of *C. merolae*, it was possible to classify the sequences to their protein family with very strong support values for Sly1p (1.0/100%/100%, Bayesian posterior probability/ML/ML corrected bootstrap values), Sec1p (1.0/95%/100%), Vps33p (1.0/100%/100%) and Vps45p (1.0/98%/100%). For *Giardia*, it was possible to assign Sly1p (1.0/60%/87%), Vps33p (1.0/89%/100%) and Vps45p (0.97/25%/85%) homologues with confidence. The putative Sec1p homologue was only supported by Bayesian posterior probabilities (0.90) and BLAST, but not bootstrap support, and hence we can only tentatively assign this sequence as a Sec1p. This is contrary to previous reports suggesting loss in *G. intestinalis *and *C. merolae *of some SM protein homologues [27] and contrary to other membrane trafficking machinery that appears reduced in these taxa [27,34].

These data strongly imply that the SM protein families originated via gene duplications from an ancestral SM protein gene, and this process must have occurred prior to the last common ancestor of the taxa sampled, which should represent a good approximation of the LCEA [19]. Our results confirm and extend previous analyses based on sparser taxon sampling [29]. Thus we see the same major patterns of acquisition of complexity *via *paralogous gene duplication as observed for the syntaxins, vesicle coats, Rabs and indeed much of the major membrane trafficking machinery. Our analyses also allow us to infer how different modes of syntaxin binding may have arisen for Vps33p. Because the HOPS complex appears to have evolved independently from the other tether complexes, and Vps33p is the only SM protein incorporated into a tether as well as the only SM protein not to interact directly with its SNARE, we deduce that Vps33p likely did ancestrally bind its syntaxin directly and may have been co-opted later by HOPS, changing its binding mode. This also implies that the ancestral mode of SM-syntaxin interaction was through direct binding.

## Conclusion

Most of the specific events within the intracellular transport system of eukaryotic cells exploit a common core of protein factors that mediate membrane budding, translocation and docking/fusion. Members of the Rab, ARF, SNARE and coat protein families participate in many of these events, and each family is clearly derived from a single common ancestor. The presence of this near universal core group of proteins mediating the basic steps of exocytosis and endocytosis indicates expansion and functional diversification prior to the formation of the major eukaryotic super-groups [24]. Here we addressed the evolutionary origins of the SM proteins as well as the tethering factor complexes, the latter having a substantially more diverse structural basis than GTPases and SNAREs.

By comparisons of the complete genomes of 17 taxa representing five of the six major eukaryotic groups [19] for 40 tethering factors and the SM proteins, we determined that both are widely distributed across eukaryotic evolution. The most likely interpretation of our findings is that these complexes are an ancient feature of the eukaryotes and have an origin that predates the diversification of the separate eukaryotic lineages. These findings further confirm the earlier indications of a complex endomembrane system for LCEA [24,27].

Lineages that lack entire tethering complexes likely lost these factors as a result of selective pressure. Multiple samplings of taxa in several major groups argue against simple failure of BLAST routines to detect these factors. Whether the failure to detect many of the tether subunits is due to methodological failure, based on the extreme sequence divergence of the homologue in question, or whether it is due to true loss, may be difficult to determine. Notwithstanding this, there is clearly a distinct pattern of conservation between these two components of the specificity machinery, i.e. the poorly conserved tether complexes and the more easily detectable and more highly conserved SM proteins. This suggests that, while there is flexibility or relaxed selection for the tethering machinery, there is more restrictive functional selection and resulting evolutionary constraints on the SM proteins. Our data also provide a guide for studies intended to probe functionality of trafficking pathways in divergent systems. For example, determining essentiality and function of incompletely retained complexes would provide an excellent means by which to test both conservation of function and the importance of retaining composition. Additionally, several of the divergent organisms are highly important pathogens, and these data provide further insights into the molecular cell biology of these systems.

Two major patterns have emerged concerning the evolution of membrane trafficking. The first pattern addresses the timing of this evolutionary innovation; multiple lines of evidence suggest that the complement of protein trafficking machinery and the organelles commonly held as involved in membrane-trafficking were established very early in eukaryotic evolution [11-14,19,22-26]. Our analyses of the tethering complexes and the SM proteins are consistent with this paradigm and add two further components to the list of characteristics possessed by the ancestral eukaryote.

The second pattern regards the process by which the machinery increased in complexity. Analyses of SNAREs [10-12], Rabs [13] and the coat proteins [14] all suggest the presence of a single ancestor of each protein family giving rise to the different organelle specific protein machineries via gene duplication. Evidence that the ARF/Sar1 family arose first, and was followed by the Rab/Ras family has also been presented [52], but our present analysis is not able to confirm or refute the concept that the ER predates the origins of the remaining endomembrane compartments. Because each of the subfamilies (or in the case of the coats, each distantly homologous coat) is characteristically associated with a particular organelle or pathway, one model is that the gene duplications were concurrent with, and possibly involved in, the process of evolutionarily deriving the various novel organelles. While the SM proteins conform precisely to this pattern, the tether complexes do not. The tethers then bring to light a novel mechanism of evolutionary elaboration of the membrane trafficking system, whereby at least some organelle specific machinery originated independently, and yet still before the LCEA.

The precise origin of the tethering complexes is somewhat equivocal, as evidence is not compelling for common ancestry amongst these factors. Even if the COG, exocyst, Dsl1 and GARP complexes were derived from an ancestral complex, the TRAPP and HOPS complexes appear independently derived. If the former four complexes did originate from a single ancestral complex, then these would have been sufficient to service the basic trafficking pathways. The latter two complexes would be later additions; however they still arose before the LCEA. In the more extreme scenario, the tether complexes each evolved convergently, with the only major common requirement being the presence of a coiled-coil forming domain that likely could interact with additional factors of the fusion machinery and the organellar membrane. As more sensitive algorithms for deducing homology become available, it may be possible to confirm or refute the relatedness of the tethering complexes and distinguish between these scenarios.

Various authors have proposed that the key to the evolution of the membrane-trafficking organelles and system are the SNAREs [11], Rabs [3] or the SM proteins [29]. The tethering complexes are the components of the specificity machinery that link all of these factors together either physically, functionally or both. An understanding of all of these components will be required to determine how vesicle fusion specificity evolved. Because the details of how the tethering complexes and other factors encode specificity remain unclear, it is too early to fully address the evolution of membrane trafficking specificity. Once a solid functional mechanism is established in model systems, determining which aspects of these mechanisms are generalisable will be the next step for elucidating a common functional mechanism and obtaining a full understanding of its evolution.

## Methods

### Databases

We selected taxa that would provide a wide sampling of the six eukaryotic super-groups, and as far as possible include at least two representative taxa in each group to facilitate detection of species-specific secondary losses versus absence from the group, and to minimise failures due to species-specific sequence divergence. In fact, we are only able to sample five supergroups as no genome sequence data are available for a representative of the Rhizaria. We also restricted our analysis to those genomes that were completed, such that failure to retrieve a BLAST hit could be ascribed to true absence or extreme divergence, and not to database incompleteness; this resulted in selection of seventeen taxa, for a total of over 650 individual BLAST queries. Trypanosomatid data were obtained from the Sanger Institute website, and either interrogated via the geneDB WWW interface [46] or locally. Yeast data were obtained from MIPS [47] or the Stanford yeast genome database [48]. *P. ramorum, Thalassiosira pseudonana*, and *Chlamydomonas reinhardtii *data were from JGI [49]. *T. gondii *data were from ToxoDB [50], *T. thermophila *data were from TIGR [51], *G. intestinalis *data from GiardiaDB [52], *D. discoideum *data at GeneDB [46], *C. neoformans *data were from the Broad Institute website [53] and *C. merolae *data from the *C. merolae *BLAST server [54]. All other organismal genome data were obtained via Uniprot [55] or the NCBI BLAST interface [56].

### Taxonomic homology survey

Data were retrieved from online databases using BLAST [57]. *H. sapiens *or *S. cerevisiae *predicted protein sequences were typically used as queries, with default BLAST parameters relaxed to maximise recovery of weaker hits. In cases where these initial queries failed to recover a candidate orthologue, query sequences from a taxon more closely related to the target genome (based on relationships from [19]) were used for further searching, should such a clear orthologue query be available, derived either from this survey or from annotated databases. All recovered sequences were subjected to reverse BLAST, typically against the *S. cerevisiae *or *H. sapiens *genome. In addition we also subjected sequences to searches through the NCBI conserved domain database (CDDB), using default parameters. This approach can detect structural relationships using alignment against HMM profiles, and while a hit is good evidence for structural (and hence sequence) relatedness, failure to retrieve a CDDB profile is not strong evidence for absence as weaker relationships may fail to be detected. For retrieval of SM candidate orthologues, *A. thaliana *or *H. sapiens *orthologues were used, with candidate orthologues validated by reverse BLAST against the nonredundant protein database. Paralogue-specific annotation was derived from phylogenetic analysis described below.

A candidate orthologue was considered to have been retrieved if a reverse BLAST recovered the original query within the top five hits. Additionally, both for initial candidate identification and for validation by reverse BLAST, sequences were analysed by alignment for the presence of significant sequence similarities, and also were parsed through NCBI CDDB rather than relying solely on e-value cut-offs. Failure to complete all of these tests resulted in an assignment of "not found".

### Inter- and intra-complex homology assessment

With the exception of the relationships between some of the TRAPP subunits, BLAST alone failed to identify sequence relationships between the tethering factors. In order to increase sensitivity, PSI-BLAST analysis was undertaken. Three iterations were performed against the NCBI nr database using *S. cerevisiae *or metazoan sequences as queries. All hits retrieved above the default threshold were inspected, and a relationship was only considered valid if factors from multiple diverse taxa were returned, and if there was evidence for a reciprocal relationship, i.e. factor A identified factor B, and *visa versa*. Additional alignments and reverse BLAST experiments were performed to verify relationships as appropriate.

### Alignments and phylogenetic reconstructions

Initial alignments of the tethering factors and the SM proteins were created in Clustal X [58] and manually adjusted. For the SM proteins, taxa were then added by hand from pair-wise Clustal alignments of the relevant taxon with the nearest representative within each eukaryotic supergroup. Several alignments were constructed. A template alignment with a single taxonomic representative of each major eukaryotic super-group for each SM protein family was created with 32 taxa and 326 positions. An alignment with all taxa was then created with 80 taxa and 318 positions. A dataset with the long branch taxa, *G. intestinalis *and *C. merolae*, removed was then made (72 taxa and 282 positions). Finally the *G. intestinalis *and *C. merolae *sequences were added back to the template alignment producing alignments with 36 taxa and 338 and 336 positions respectively. Analysis of these final two alignments enabled classification of the SM protein representatives from these divergent taxa. Since the purpose of the study was to establish whether each taxon had at least one representative of each SM protein family, and not to fully resolve the classification of each protein from each taxon sampled, some homologues from some taxa were excluded from the phylogeny. Only the most canonical homologue of each protein family, as assessed by BLASTp score and by size comparison, was used for the relevant phylogeny. All alignments were masked such that only unambiguously homologous positions were used for phylogenetic analysis and are available upon request. The model of sequence evolution for each dataset was determined using Tree-Puzzle v.5.2 [59] based on initial neighbor-joining trees and incorporating an 8-category gamma correction for rate variation. Trees were then built using Mr. Bayes v. 3.1.2 [60] for Bayesian analysis to determine optimal tree topology and posterior probability values for the nodes, with 1 000 000 Markov Chain Monte Carlo generations and the burn-in value determined graphically by removing trees before the plateau. Phyml v.2.4.4 [61] was used to obtain maximum-likelihood bootstrap values; and Fitch or Neighbor-Joining v.3.6a3 from the PHYLIP package [62] using the distance matrices generated by Tree-Puzzle and Puzzleboot [63] from 1000 (for Neighbor-Joining) or 100 (for Fitch) pseudo-replicate datasets respectively. Nodes with greater than 0.95 posterior probability and better than 80% bootstrap support were considered robust, although in Figure 5 all nodes with support values greater than 0.95 posterior probability and 50% bootstrap are shown. Other than the template alignment (32 taxa, 318 positions), which was analyzed by ML and ML-corrected distance only, all alignments were analyzed by all three methods.

## Abbreviations

COG, conserved oligomeric complex; GARP, Golgi associated retrograde protein complex; HOPS, homotypic fusion and vacuole protein sorting; LCEA, last common eukaryotic ancestor; ML, maximum likelihood; ORF, open reading frame; SM, Sec1/Munc18; SNARE, soluble NSF-attachment factor receptor; TRAPP, transport protein particle.

## Authors' contributions

VLK, JBD and MCF were responsible for conceiving and designing the study. VLK and MCF performed the analyses on the tether complexes, and JBD did the analysis of the SM proteins. RMRC processed BLAST results for graphical representation. All authors were responsible for drafting the manuscript and producing the figures.

## Supplementary Material

Additional File 1 Coiled-coil domain annotations and regions of sequence similarity for selected tethering factors. Coiled-coil predictions were done using Coils, with default settings [65]. Regions of sequence similarity returned by PSI-BLAST are indicated by a grey bar for each prediction. Sequences are truncated at 800 residues for comparative purposes. Click here for file

Additional File 3 Distribution of tethering complex subunits across representative eukaryotic taxa by BLAST. Data are based on BLAST results together with alignments – typically the *S. cerevisiae *or *H. sapiens *sequences were used as queries. Y = an identification based on a clear reverse BLAST result and/or additional evidence through analysis of the sequence by Clustal. Names of individual factors are given for *S. cerevisiae*, with synonyms following, if applicable. N = not found. Footnotes: 1; The *C. reinhardtii *genome is fragmentary at this time and in some instances BLAST retrieves only short sequences that can be defined by domains only and not the full length ORF. 2; COG5 and COG7 lack conserved domains as detected in CDDB – and are less well conserved between yeast and humans; hence these subunits may be particularly difficult to identify explaining their absence from several genomes. 3; Vps51 is a small ORF and therefore less likely to be found due to fewer possible sites for identification and the higher probability of it not being sequenced in a random sequencing approach. 4; Reverse BLAST to *S. cerevisiae *Sec5p with e^-6 ^but not to *H. sapiens *or other taxa. Given absence of remaining orthologues, status is equivocal. 5; Reverse BLAST to Viridiplantae Sec6p with e^-7 ^but not other taxa. Contains 40% of Sec6 domain, e^-9 ^by CD search, therefore probably a truncated form. 6; Weak reverse BLAST, but all contain part of Sec20 domain. Equivocal status. 7; *H. sapiens *homologue, identified by Bet3p pull down, is twice the molecular weight of *S. cerevisiae *Trs85p and only shows weak similarity by BLAST. Most other candidates recovered using *H. sapiens *query. *C. elegans *has two isoforms which do reverse BLAST, albeit weakly, to *S. cerevisiae*. Click here for file

Additional File 2 Clustal X alignment of Bet5p orthologues from selected taxa. Full length predicted amino acid sequences from selected taxa were aligned using Clustal X. Dashes indicated gaps introduced to improve the alignment. A "*" on the consensus line indicates fully conserved residues, ":" indicates conservative substitutions, and "." indicates conservation in 50% of taxa. Click here for file
