# Supplementary figures and images for: Control systems for membrane fusion in the ancestral eukaryote; evolution of tethering complexes and SM proteins
Source: BMC Evol Biol. 2007 Feb 23;7:29. doi: 10.1186/1471-2148-7-29 (PMC1810245; doi:10.1186/1471-2148-7-29)

Coil probability/arbitrary units

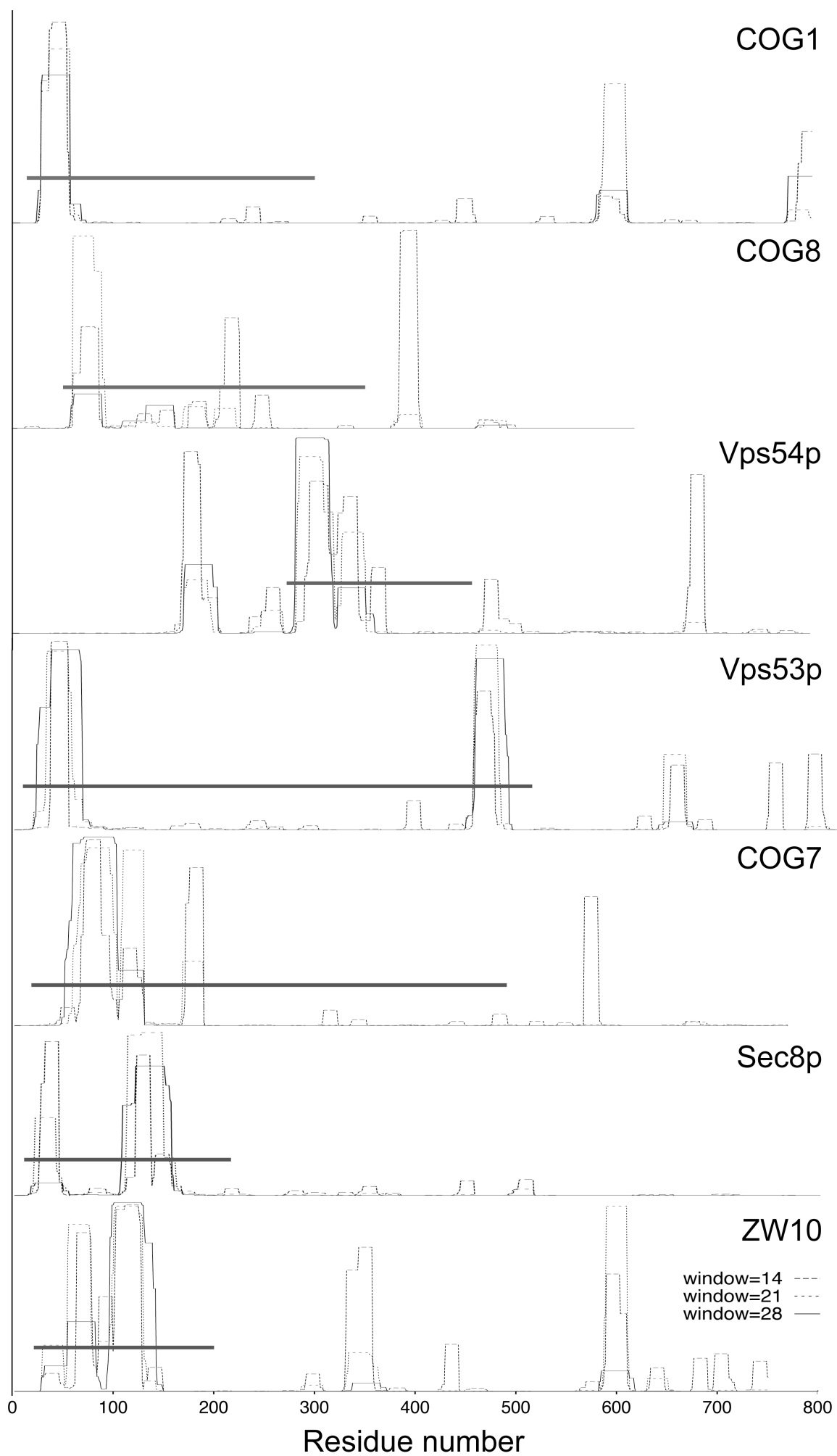

Supplement: Additional File 1 — Coiled-coil domain annotations and regions of sequence similarity for selected tethering factors. Coiled-coil predictions were done using Coils, with default settings [65]. Regions of sequence similarity returned by PSI-BLAST are indicated by a grey bar for each prediction. Sequences are truncated at 800 residues for comparative purposes. [file 1471-2148-7-29-S1.pdf]
