# Supplementary material for: Control systems for membrane fusion in the ancestral eukaryote; evolution of tethering complexes and SM proteins
Source: BMC Evol Biol. 2007 Feb 23;7:29. doi: 10.1186/1471-2148-7-29 (PMC1810245; doi:10.1186/1471-2148-7-29)
Supplement: Additional File 3 — Distribution of tethering complex subunits across representative eukaryotic taxa by BLAST. Data are based on BLAST results together with alignments – typically the S. cerevisiae or H. sapiens sequences were used as queries. Y = an identification based on a clear reverse BLAST result and/or additional evidence through analysis of the sequence by Clustal. Names of individual factors are given for S. cerevisiae, with synonyms following, if applicable. N = not found. Footnotes: 1; The C. reinhardtii genome is fragmentary at this time and in some instances BLAST retrieves only short sequences that can be defined by domains only and not the full length ORF. 2; COG5 and COG7 lack conserved domains as detected in CDDB – and are less well conserved between yeast and humans; hence these subunits may be particularly difficult to identify explaining their absence from several genomes. 3; Vps51 is a small ORF and therefore less likely to be found due to fewer possible sites for identification and the higher probability of it not being sequenced in a random sequencing approach. 4; Reverse BLAST to S. cerevisiae Sec5p with e-6 but not to H. sapiens or other taxa. Given absence of remaining orthologues, status is equivocal. 5; Reverse BLAST to Viridiplantae Sec6p with e-7 but not other taxa. Contains 40% of Sec6 domain, e-9 by CD search, therefore probably a truncated form. 6; Weak reverse BLAST, but all contain part of Sec20 domain. Equivocal status. 7; H. sapiens homologue, identified by Bet3p pull down, is twice the molecular weight of S. cerevisiae Trs85p and only shows weak similarity by BLAST. Most other candidates recovered using H. sapiens query. C. elegans has two isoforms which do reverse BLAST, albeit weakly, to S. cerevisiae. [file 1471-2148-7-29-S3.pdf]

Tethers Master

		**Opistokonta**				**Amoebozoa**	**Plantae**			**Chromalveolata**					**Excavata**			
							Viridiplantae		Rhodophyta	Alveolata			Stramenopiles					
		Fungi		Metazoa						Apicomplexa		Ciliates			Kinetoplastida			Diplomonads
**COMPLEX**	**FACTOR**	*S. cerevisiae*	*C. neoformans*	*H. sapiens*	*C. elegans*	*D. discoidium*	*A. thaliana*	*C. reinhardtii*[1]	*C. merolae*	*P. falciparum*	*T. gondii*	*T. thermophila*	*P. ramorum*	*T. pseudonana*	*T. brucei*	*T. cruzi*	*L. major*	*G. intestinalis*
	**Name, synonym1, synonym2, etc**																	
**HOPS**	Vps11, Pep5, End1	Y	Y	Y	Y	Y	Y	Y	N	Y	Y	Y	Y	Y	Y	Y	Y	Y
	Vps16	Y	Y	Y	Y	Y	Y	Y	N	Y	Y	Y	Y	Y	Y	Y	Y	N
	Vps18, Pep3	Y	Y	Y	Y	Y	Y	N	N	Y	Y	Y	Y	N	Y	Y	Y	N
	Vps33, Slp1, Vam5	Y	Y	Y	Y	Y	Y	Y	Y	Y	Y	Y	Y	Y	Y	Y	Y	Y
	Vps39, Vam6	Y	N	Y	Y	Y	Y	N	N	N	N	N	N	N	Y	Y	Y	N
	Vps41, Vam2	Y	Y	Y	Y	Y	Y	Y	N	N	N	N	Y	Y	Y	Y	Y	Y
**COG**	COG1, LDLB, Sec36	Y	N	Y	Y	Y	Y	N	N	N	N	N	Y	N	Y	Y	Y	N
	COG2, LDLC, Sec35	Y	N	Y	Y	Y	Y	Y	Y	N	N	N	Y	Y	Y	Y	Y	N
	COG3, Sec34, Grd20	Y	Y	Y	Y	Y	Y	Y	Y	N	Y	Y	Y	Y	Y	Y	Y	N
	COG4, Sec38	Y	Y	Y	Y	Y	Y	Y	Y	N	Y	Y	Y	N	Y	Y	Y	N
	COG5, Tfi3, GTC-90[2]	Y	Y	Y	N	Y	Y	Y	N	N	N	N	Y	Y	Y	Y	Y	N
	COG6, hCod2	Y	N	Y	Y	Y	Y	Y	N	N	N	N	Y	Y	Y	Y	Y	N
	COG7, hCod5, Sec37[2]	Y	N	Y	N	Y	Y	Y	N	N	N	N	Y	N	Y	Y	Y	N
	COG8, Dor1, Cod5	Y	N	Y	Y	Y	Y	Y	Y	N	N	N	Y	Y	Y	Y	Y	N
**GARP**	Vps51, API3, WHI6, Vps67[3]	Y	N	Y	N	N	N	N	N	N	N	N	N	N	N	N	N	N
	Vps52, Sac2, ARE1, SACM2L	Y	Y	Y	Y	Y	Y	Y	N	N	Y	Y	N	Y	Y	Y	Y	N
	Vps53	Y	Y	Y	Y	Y	Y	N	Y	Y	Y	Y	N	Y	Y	Y	Y	N
	Vps54	Y	Y	Y	N	N	Y	N	N	N	N	N	N	N	Y	Y	Y	N
**Exocyst**	Sec3, EXOC1	Y	Y	Y	Y	Y	Y	N	N	N	N	N	N	Y	Y	Y	Y	N
	Sec5, EXOC2	Y	Y	Y	Y	Y	Y	Y	N	Y[4]	N	N	N	Y	N	N	N	N
	Sec6, EXOC3	Y	Y	Y	Y	Y	Y	Y	Y[5]	N	N	Y	N	N	Y	Y	Y	N
	Sec8, EXOC4	Y	Y	Y	Y	Y	Y	Y	N	N	Y	N	Y	N	Y	Y	Y	N
	Sec10, EXOC5	Y	Y	Y	Y	Y	Y	N	N	N	N	Y	Y	N	Y	Y	Y	N
	Sec15, EXOC6	Y	Y	Y	Y	Y	Y	N	N	N	N	Y	Y	N	Y	Y	Y	N
	Exo70, EXOC7	Y	Y	Y	Y	Y	Y	Y	N	N	N	N	Y	Y	Y	Y	Y	N
	Exo84, EXOC8	Y	Y	Y	Y	Y	Y	Y	N	N	N	N	N	N	N	N	N	N
**Dsl1**	Dsl1, ZW10	Y	N	Y	Y	Y	Y	N	N	N	N	N	Y	Y	N	N	N	N
	Dsl3, Sec39	Y	N	N	N	N	N	N	N	N	N	N	N	N	N	N	N	N
	Tip20, RINT1	Y	Y	Y	N	Y	Y	Y	N	N	N	N	Y	N	N	N	N	N
	Sec20, BNIP1	Y	Y	Y	N	Y	Y	Y	N	Y	N	N	N	Y	Y[6]	Y[6]	Y[6]	Y
**TRAPPI**	Trs20, TRAPPC2, SEDL	Y	Y	Y	Y	Y	Y	Y	Y	Y	Y	Y	Y	Y	Y	Y	N	N
	Trs23, TRAPPC4, synbindin	Y	Y	Y	Y	Y	Y	Y	Y	Y	Y	N	Y	Y	Y	Y	Y	Y
	Trs31, TRAPPC5	Y	Y	Y	Y	Y	Y	Y	Y	Y	Y	Y	Y	Y	Y	Y	Y	Y
	Trs33, TRAPPC6A	Y	Y	Y	Y	Y	Y	Y	Y	Y	Y	Y	Y	Y	Y	Y	Y	N
	Trs85, Gsg1, Mum1, TRAPPC8[8]	Y	Y	Y	Y	Y	Y	Y	Y	Y	Y	Y	Y	Y	Y	Y	Y	N
	Bet3, TRAPPC3	Y	Y	Y	Y	Y	Y	Y	Y	Y	Y	Y	Y	Y	Y	Y	Y	Y
	Bet5, TRAPPC1	Y	Y	Y	Y	Y	Y	Y	Y	Y	Y	Y	Y	Y	Y	Y	Y	Y
**TRAPPII**	Trs65, Kre11, TRAPPC7	Y	N	N	N	N	N	N	N	N	N	N	N	N	N	N	N	N
	Trs120, TRAPPC9	Y	N	Y	Y	Y	Y	Y	N	N	N	Y	Y	Y	N	N	N	N
	Trs130, TRAPPC10	Y	Y	Y	Y	Y	Y	N	N	N	N	N	Y	Y	N	N	N	N
