# Supplementary material for: Control systems for membrane fusion in the ancestral eukaryote; evolution of tethering complexes and SM proteins
Source: BMC Evol Biol. 2007 Feb 23;7:29. doi: 10.1186/1471-2148-7-29 (PMC1810245; doi:10.1186/1471-2148-7-29)
Supplement: Additional File 2 — Clustal X alignment of Bet5p orthologues from selected taxa. Full length predicted amino acid sequences from selected taxa were aligned using Clustal X. Dashes indicated gaps introduced to improve the alignment. A "*" on the consensus line indicates fully conserved residues, ":" indicates conservative substitutions, and "." indicates conservation in 50% of taxa. [file 1471-2148-7-29-S2.pdf]

```
H.sapiens|Q9Y5R8|TPPC1_HUMAN        --------------------MTVHNLYLFDRNGVCLHYSEWHRKKQ---- 26
A.thaliana|NP_175528.1|unknown      MQFFGGSEISPSPPVPTASGNNAHMMYVFNRNGVCLLYKEWNRPLH---- 46
C.elegans|NP_001023758.1|           --------------------MTIYNVYIFDREGQCLYYDEWFRTKQ---- 26
C.elegans|NP_001023759.1|           --------------------MTIYNVYIFDREGQCLYYDEWFRTKQ---- 26
S.cerevisiae|NP_013634.1|Bet5p      --------------------MGIYSFWIFDRHCNCIFDREWTLASNSASG 30
P.falciparum|PF14_0049|||hypot      ----MILSNEKIPQVTDKWGDMYYYFYIFYKN-QCIYSIDLKNNNTQEKK 45
                                                           : .::* :.  *:   :

H.sapiens|Q9Y5R8|TPPC1_HUMAN        --AGIPKEEEYKLMYGMLFSIRSFVSKMSPL------------------- 55
A.thaliana|NP_175528.1|unknown      --TLNP-QQDHKLMFGLLFSLKSLTAKMDPV------------------- 74
C.elegans|NP_001023758.1|           --SGLAPIQEYKLVFGMMLSMKSFVDRLATN------------------- 55
C.elegans|NP_001023759.1|           --SGLAPIQEYKLVFGMMLSMKSFVDRLATN------------------- 55
S.cerevisiae|NP_013634.1|Bet5p      TINSKQNEEDAKLLYGMIFSLRSITQKLSKG------------------- 61
P.falciparum|PF14_0049|||hypot      --KKSNKNETEKLLLGSIYAINYLCSNIQPNKKLKNLYKSISNSNPKLIN 93
                                            :  **: * : ::. :  .:

H.sapiens|Q9Y5R8|TPPC1_HUMAN        -DMKDG-------------FLAFQTSRYKLHYYETPTGIKVVMNTDLGVG 91
A.thaliana|NP_175528.1|unknown      -NADKGNLGVPQLPGQGCSFHSFRTNTYKLSFMETPSGIKIILVTHPKTG 123
C.elegans|NP_001023758.1|           -DSNQT-------------VNYYKTSAYKMTFLESATSIKIMLNTDPNAT 91
C.elegans|NP_001023759.1|           -DSNQT-------------VNYYKTSAYKMTFLESATSIKIMLNTDPNAT 91
S.cerevisiae|NP_013634.1|Bet5p      -SVKND-------------IRSISTGKYRVHTYCTASGLWFVLLSDFKQQ 97
P.falciparum|PF14_0049|||hypot      TNIQTQNNINTQENIHVGNFNCFNTPFYKLHYVETLTAYKFVLITHKNIP 143
                                     . .               .    *  *::    : :.  .:: :.

H.sapiens|Q9Y5R8|TPPC1_HUMAN        PIRDVLHHIYSALYVELVVKNPLCPLGQTVQSELFRS------------- 128
A.thaliana|NP_175528.1|unknown      DLRESLKYIYS-LYVEYVVKNPIYSPGSPIKSELFNT------------- 159
C.elegans|NP_001023758.1|           GIRDLLHKIYQTWTETANSAAKLELFASNPPNENFLKNHKKMSEPSAPPD 141
C.elegans|NP_001023759.1|           GIRDLLHKIYQTWTETANSAAKLELFASNPPNENFLKNHKKMSEPSAPPD 141
S.cerevisiae|NP_013634.1|Bet5p      SYTQVLQYIYSHIYVKYVSNNLLSPYDFAENENEMRG------------Q 135
P.falciparum|PF14_0049|||hypot      NLSNFLKDIYKTIFIDLIILNPVYKIGDEIKDKMFDE------------- 180
                                       : *: **.           :        .: :

H.sapiens|Q9Y5R8|TPPC1_HUMAN        RLDSYVRS--LPFFSARAG------------------------------- 145
A.thaliana|NP_175528.1|unknown      ALDQYVRS--IS-------------------------------------- 169
C.elegans|NP_001023758.1|           EAESTIRSPDLPFVSSIVGGLFAGRAIVVSGMVLPGFASDRKRFQIDLCC 191
C.elegans|NP_001023759.1|           EAESTIRSPDLPFVSSIVGGLFAGRAIVVSGMVLPGFASDRKRFQIDLCC 191
S.cerevisiae|NP_013634.1|Bet5p      GTRKITNRNFISVLESFLAPMVNQ-------------------------- 159
P.falciparum|PF14_0049|||hypot      KILEKIKRLYVG-------------------------------------- 192
                                       .  .   :

H.sapiens|Q9Y5R8|TPPC1_HUMAN        --------------------------------------------------
A.thaliana|NP_175528.1|unknown      --------------------------------------------------
C.elegans|NP_001023758.1|           GLLIDGDHMDNKALHFNPRFDAKTGWFSGPGDDKLVINSFVSGRWGNEER 241
C.elegans|NP_001023759.1|           GLLIDGDHMDNKALHFNPRFDAKTGWFSGPGDDKLVINSFVSGRWGNEER 241
S.cerevisiae|NP_013634.1|Bet5p      --------------------------------------------------
P.falciparum|PF14_0049|||hypot      --------------------------------------------------

H.sapiens|Q9Y5R8|TPPC1_HUMAN        --------------------------------------------------
A.thaliana|NP_175528.1|unknown      --------------------------------------------------
C.elegans|NP_001023758.1|           FDNPFKEGEPFQIRIMVFEKYFKISASGKHMCDFPHRVPVESIRTISING 291
C.elegans|NP_001023759.1|           FDNPFKEGEPFQIRIMVFEKYFKISASGKHMCDFPHRVPVESIRTISING 291
S.cerevisiae|NP_013634.1|Bet5p      --------------------------------------------------
P.falciparum|PF14_0049|||hypot      --------------------------------------------------

H.sapiens|Q9Y5R8|TPPC1_HUMAN        --------------------------------------------------
A.thaliana|NP_175528.1|unknown      --------------------------------------------------
C.elegans|NP_001023758.1|           NIRVDYVEFHPPE----DSMPTE--------------------------- 310
C.elegans|NP_001023759.1|           NIRVDYVEFHPPIGIGADGKPTLVAPTPKQEVITKIDKPNVPFELPLPPG 341
S.cerevisiae|NP_013634.1|Bet5p      --------------------------------------------------
```

```
P.falciparum|PF14_0049|||hypot      --------------------------------------------------


H.sapiens|Q9Y5R8|TPPC1_HUMAN        --------------------------------------------------
A.thaliana|NP_175528.1|unknown      --------------------------------------------------
C.elegans|NP_001023758.1|           --------------------------------------------------
C.elegans|NP_001023759.1|           GFVSPQSARFTITPFLSSERFTINLKSKGEFLFHFRVDMPNQAQKIKPHV 391
S.cerevisiae|NP_013634.1|Bet5p      --------------------------------------------------
P.falciparum|PF14_0049|||hypot      --------------------------------------------------


H.sapiens|Q9Y5R8|TPPC1_HUMAN        --------------------------------------------------
A.thaliana|NP_175528.1|unknown      --------------------------------------------------
C.elegans|NP_001023758.1|           --------------------------------------------------
C.elegans|NP_001023759.1|           IRNSSKNGVKWLTEERTFGTFPFHKGITHDIVFTAYGKSVTVDVDGAPFV 441
S.cerevisiae|NP_013634.1|Bet5p      --------------------------------------------------
P.falciparum|PF14_0049|||hypot      --------------------------------------------------


H.sapiens|Q9Y5R8|TPPC1_HUMAN        -------------------------------
A.thaliana|NP_175528.1|unknown      -------------------------------
C.elegans|NP_001023758.1|           -------------------------------
C.elegans|NP_001023759.1|           KFVYRDGDDPVNVDQITVVGDVLIHRFEHKG 472
S.cerevisiae|NP_013634.1|Bet5p      -------------------------------
P.falciparum|PF14_0049|||hypot      -------------------------------
```
